# Expression of Multiple Sexual Signals by Fathers and Sons in the East-Mediterranean Barn Swallow: Are Advertising Strategies Heritable?

**DOI:** 10.1371/journal.pone.0118054

**Published:** 2015-02-13

**Authors:** Yoni Vortman, Rebecca J. Safran, Tali Reiner Brodetzki, Roi Dor, Arnon Lotem

**Affiliations:** 1 Department of Evolutionary and Environmental Biology, University of Haifa, Haifa, Israel; 2 Department of Zoology, Faculty of Life Sciences, Tel-Aviv University, Tel-Aviv, Israel; 3 Department of Ecology and Evolutionary Biology, Univ. of Colorado at Boulder, Boulder, CO, United States of America; 4 Fuller Evolutionary Biology Program, Cornell Lab of Ornithology, Ithaca, NY, United States of America; University of Milan, ITALY

## Abstract

The level of expression of sexually selected traits is generally determined by genes, environment and their interaction. In species that use multiple sexual signals which may be costly to produce, investing in the expression of one sexual signal may limit the expression of the other, favoring the evolution of a strategy for resource allocation among signals. As a result, even when the expression of sexual signals is condition dependent, the relative level of expression of each signal may be heritable. We tested this hypothesis in the East-Mediterranean barn swallow (*Hirundo rustica transitiva*), in which males have been shown to express two uncorrelated sexual signals: red-brown ventral coloration, and long tail streamers. We show that variation in both signals may partially be explained by age, as well as by paternal origin (genetic father-son regressions), but that the strongest similarity between fathers and sons is the relative allocation towards one trait or the other (relative expression index), rather than the expression of the traits themselves. These results suggest that the expression of one signal is not independent of the other, and that genetic strategies for resource allocation among sexual signals may be selected for during the evolution of multiple sexual signals.

## Introduction

Like most traits, the expression of sexually selected traits is affected by genes, environment, and by their interaction [1–3]. However, in sexual signals that are condition dependent, gene-environment interactions may also be viewed as the outcome of an adaptive advertising strategy that invests currently available resources towards the development and expression of a costly signal [4–8]. Importantly, under this view, the heritable trait that is under selection may not be the absolute size or intensity of the signal, but rather the strategy for allocating resources towards signal development and expression.

Advertising strategies may be especially important for animals that use multiple sexual signals [5,9,10], as they are expected to trade off their investment between different signals [5,11–15]. The existence of such a trade-off is supported by studies that found negative correlations between phenotypic expression of sexually selected traits [11,16]. However, trade-offs may not necessarily produce negative correlations. For example, female preference for high expression of multiple sexual signals may drive a positive correlation between signals in cases where high quality males are capable of developing and expressing extreme forms of both signals [5]. Additionally, negative correlations between signal expressions may be genetically based [15]. This possibility implies that different individuals have different genetic strategies for resource allocation among signals. In this case, the relative investment in each signal can be heritable even if their absolute magnitude may not be. It should be noted, however, that heritable advertising strategies can also produce positive correlations between signals, or no correlation at all if their genetic basis is masked by phenotypic variation in male quality or condition. To clarify this issue, in the Supplementary Information (Table A and Figure A in S1 File) we demonstrate a hypothetical situation in which an advertising strategy for resource allocation between two signals can be perfectly heritable (i.e. identical for father and son) but, nevertheless, the expression of each signal shows no father-son similarity and no correlation exists between the two signals. Considering such a wide range of possibilities, a good way to identify heritable advertising strategies would be to study their heritability directly in species that uses multiple sexual signals. This can be done by comparing the relative expression of different signals by fathers and sons in a species that uses multiple sexual signals. To the best of our knowledge, however, despite recent recognition of the role of genetic trade-offs between the expression of multiple sexual traits [15], such an analysis has never been carried out.

We studied the effects of genetic origin and ontogenetic variables (age and condition in the nest) on the expression of multiple sexual signals in the East-Mediterranean barn swallow (*H. r. transitiva*). The barn swallow’s long tail streamers and dark ventral coloration have served as a classic model system for studying sexually selected traits [17–27]. Both tail streamer length and ventral coloration have been shown to be both heritable [28–30] and condition-dependent [28,25,26,31] in the European population.

The East-Mediterranean population (*H. r. transitiva*) is considered a sedentary population of barn swallows [19,32], which breeds along the east coast of the Mediterranean, south of the distribution range of the migratory European population (*H. r. rustica*) and north of the sedentary Egyptian population (*H. r. savignii*). The East-Mediterranean population (*H. r. transitiva*) exhibits an intermediate combination of the two above-mentioned sexual signals, having both long tail streamers and dark ventral coloration [33,34], and both ornaments play a role in mate choice in this population [33,32]. The intermediate expression of both signals in the *H. r. transitiva* population, as opposed to the strong expression of only one of them in the other, above-mentioned, neighboring populations, suggests that the production and/or maintenance of these two traits simultaneously may be too costly, favoring adaptive strategy for resource allocation between them (see also [35], [36] for evidence for the cost of tail streamers and ventral coloration in the European and North American populations, respectively).

By comparing the absolute and relative levels of expression of these ornaments in the same individuals over several years, and using genetic father-son regressions, we assessed age and genetic effects on ornament expression. We hypothesized that the variation in both ventral coloration and tail streamer length would have a significant heritable component and that both traits’ expression would also be affected by age and individual condition. Further, by developing an index for the relative expression of the two signals and testing its similarity between fathers and their sons, our analysis also allowed us to examine the idea of a genetic resource allocation strategy between concurrent costly multiple sexual signals.

## Methods

### Ethical standards

This study was conducted according to Tel-Aviv University guidelines and under annual permits from the Israeli Nature and Parks Authority for capturing, handling and sampling blood and feathers from barn swallows (permit numbers: 28234–2007, 31345–2008, 32105–2009 and 37160–2010).

### General methods

We studied a population of barn swallows at two breeding sites in Israel, from November 2006 through July 2010, covering four full breeding seasons (see also [33,32]). Breeding adults were captured and individually marked with numbered aluminum rings and passive RFID 12 mm, 0.095 g tags. All captured adults were weighed and measured for wing length, tail streamer length (outer rectrices), and the tail feathers adjacent to these streamers. For paternity assignment we took a ~ 20μl blood sample from the brachial vein of each individual and preserved each sample in 1 ml of lysis buffer with 2% SDS [37]. During the years 2007–2010 we banded and genotyped 768 nestlings. At the age of 10–12 days, all nestlings were weighed and ranked with respect to their brood based on their body mass. Of the 768 nestlings, 31 males and 14 females returned the three following years (2008–2010, spread evenly) as first or second year breeders, only males were used in this analysis. We verified that there was no effect of hatching year on the morphology of offspring born in our study area that returned as breeders with respect to tail streamer length and ventral coloration for both first and second year males (*P* > 0.4 for all). This created a valuable data set which enabled us to assess father-son resemblance of the various morphological traits by plotting the recruited males’ morphological data against the morphology of their genetic fathers [2]. Further, this also enabled us to assess the effect of known age on morphology, a valuable opportunity, as once they reach adulthood, absolute age in barn swallows is hard to determine.

### Color measurements

To assess ventral plumage color, we plucked 2–4 feathers from the ventral region (between the breast and the cloaca) of each captured bird and mounted them on an index card for further analysis, following previously established procedures [33,32]. We then photographed the feather cards for digital color analysis (See [33,32] for more details).

We applied digital photography following Stevens et al. [38], using RAW file formats and Manual White Balance. For digital image analysis and color scoring we collaboratively developed a MATLAB tool (The Mathworks Inc) with the Signal and Image Processing Lab at the Technion, which enabled us to exclude pixels with background effect [33]. Color scoring was done with the sRGB color space, scoring the feather’s chromatic elements on the R/G and G/B ratio, which is consistent with vertebrate perception of chromatic properties [39,40], and with its relative insensitivity to variations in lighting intensity [41]. We further simplified our color scoring method using the R/B (red/blue) ratio, which is sufficiently accurate as a single color score: it was perfectly correlated to a principal component of R/G and G/B (*r*_*s*_ = 0.9997, *n* = 219, *P* ~ 0.000; PC1 for R/G-G/B accounted for 98.6% of the variance, R/G, G/B loadings = 0.99284). For the analysis presented in this paper we photographed each feather card twice, removing it and then replacing it under the camera, and measured the repeatability of the R/B color score for 203 feather cards (*r* = 0.98). We used 20 feather cards that could be measured accurately (i.e. without background bias) by an Ocean Optics USB-4000 spectrometer (range 200–1100 nm, Ocean Optics Inc., Dunedin, USA), and confirmed that their chroma score was highly correlated with the R/B score obtained from the digital photographs of the same feathers (*r*_*s*_ = 0.86, *n* = 20, *P* < 0.00001). Chroma was calculated as the reflectance sum over the peak reflectance range (between 600nm-700nm) divided by the total reflectance sum (between 300nm-700nm), (see [33] for further details).

### Paternity analysis

DNA of adults and nestlings was extracted from blood samples using Qiagen 96 well-plate DN-easy blood kit. To assign paternity, we amplified seven microsatellite loci (see [33]), creating a powerful test for exclusion of social males in cases of extra-pair young within the brood (second parent exclusion probability is 0.9999). Each offspring was assigned to its genetically most likely father, and independently to the most likely parent pair considering the social mother’s genotype. Paternity assignment was done using Cervus V3 [42]. Identification of extra-pair fathers was done only when both assignments converged to the same genetic father and only when both the social mother’s and father’s genotypes were known. Out of the 31 recruited male nestlings four were sired by an extra-pair father.

### Sexing

As yearlings in nature were less ornamented (see Results) the traditional methods for non-molecular sexing (i.e. tail streamer length differences [43]) were not completely reliable for assigning sex. Thus we assigned sex using molecular tools with the P2/P8 primer set to amplify the CHD genes, following Griffiths et al. [44].

### Developing an index for the relative expression of signals

**Fig 1:**
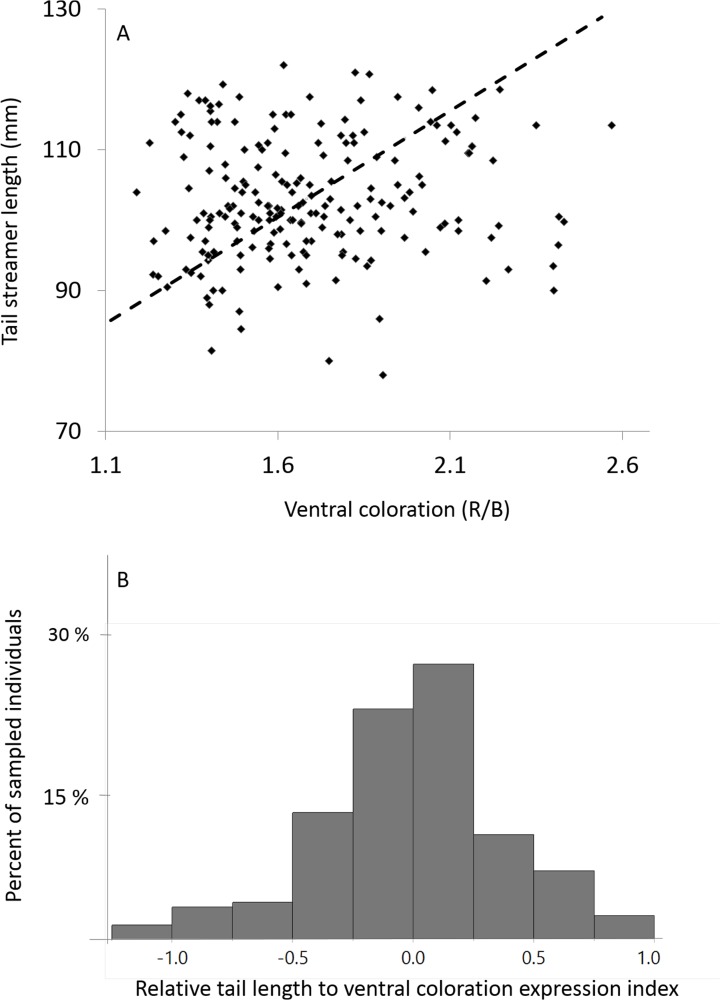
Distribution of males’ tail streamer length, ventral coloration and their relative expression index. 1A: Distribution of tail streamer length and ventral coloration measured from 200 adult males in our study population. The two traits do not correlate (*R* ^*2*^ = 0.0007, *P >* 0.23). The dashed diagonal line, determined by the two traits’ means and standard deviations, represents the population norm of “equal” relative expression of the two signals, allowing use of the minimal distance to this line as the relative expression index of the two signals (see Methods section). 1B: Distribution of the relative expression index scores, *n* = 200.

A methodological challenge lies in creating a valid index to score the relative expression of two sexual signals (tail streamer length and ventral coloration). One obvious way to create such a score is to compute the residual of one signal expression when plotted against the other (i.e. the vertical distance between a data point and the regression line ([51] p—532–533), thus representing, for example, the extent to which the expression of tail length is above or below expected relative to color darkness. The problem, however, is that this method can only work if the two traits are correlated. As noted in the introduction, multiple signals may not be correlated with each other, which is also the case in our studied population (see Fig. 1). This precluded the use of this method in our study, as well as in any other case where multiple signals are not correlated. A simple alternative is to compute the ratio between trait expression, as in the extensively used Body Mass Index (BMI) in humans (e.g. [45,46,47,48–50]). However, ratios tend to have undesirable statistical properties and their use in biological studies is somewhat controversial ([51], P-17).

To avoid the use of ratios but still retain our ability to measure the relative expression of signals, we took a geometric approach that is somewhat similar to the use of residuals but does not require that the two traits will be correlated. This index is based on the minimal (perpendicular) distance of each individual from the population diagonal line of “equal” relative expression of the two signals. To create this index we first plotted the population data points (of N = 200 males) in the two-dimensional space of the two signals (Fig. 1). We then superimposed the diagonal line of “equal” signals expression based on the two signals’ means and standard deviations (i.e. the line crossing the *x*,*y* locations of signals means and lower and upper standard deviations, see Fig. 1). This line represents the mean relative expression of the two signals in the population, which we view as the equality line in terms of the population’s norm (it may be different in different populations). Each male can then be characterized according to its distance from this line: the greater the distance, the greater the deviation from the population’s norm towards a greater relative expression of long tail or dark color. The minimal distance between a male phenotype (*x*,*y* coordinates) and the population diagonal line was calculated using the minimal distance equation:

$$\left[distance\;from\;a\;point\;x_{1},y_{1}\;to\;a\;line\;\left(ax+by+c\;=\;0\right)\right]=\frac{\left|ax_{1}+by_{1}+c\right|}{\sqrt{a^{2}+b^{2}}}$$

We assigned positive values to distances above the line and negative values to distances below the line. Finally, we verified that these new index scores followed a normal distribution (were not distributed significantly differently from normal (Kolmagorov-Smirnov test, *d* = 0.057, *p = n.s*., see also Fig. 1B)). It is important to note that the line of “equal relative expression” is a linear approximation of the population norm, used as the most parsimonious method for generating the relative expression index. It may not represent the real line of equal investment in the two traits that is affected by the relative cost of investing in each, and may not be necessarily linear.

### Father—son similarity

The similarity between fathers and sons was based on the regression of offspring on parent (see statistical methods below for further details). Since female expression of male ornaments is limited, and their genetic contribution to offspring ornamentation is not clear, we focused on father-son regressions. Further, as these two sexual signals show significant sexual dimorphism [33], we could not use mid-parent offspring regression [2]. It is important to note that we examined father-son similarity in the wild without a cross-fostering experiment. Consequently, interpreting the slope of the regression into estimates of heritability should be done with caution. Theoretically, when computed from a single-parent to offspring regression, the heritability estimate (*h* ^*2*^) should equal twice the regression slope [2]. However, as we do not aim to indicate the exact *h* ^*2*^ but, rather, the general level of heritability, we report the slope of the regression throughout. By using a mixed model including repeated measurements of sons at different ages and using the REML method, our statistical model (see below) goes beyond a simple father-son regression and minimizes potential differences with the ‘animal model’ method [52].

### Repeatability

Repeatability (R) can be defined as the proportion of the total variance accounted for by differences among individuals [51,52], and may serve as a rough upper limit estimate of *h* ^*2*^ [2,53,54]. We calculated repeatability between consecutive years “*R*” of tail streamer length, ventral coloration and their relative expression index following [51]. The standard error (*SE*) of the repeatability estimate was calculated following Becker [55] and Nakagawa and Schielzeth [52].

### Controlling for parental non-genetic effects

The phenotype of offspring born and recruited to our breeding sites was measured approximately a year or two after they had fledged, a few months following the first (or second) adult feather molt. This relatively long time-span from fledging potentially reduces the link between parental care and recruited nestling morphology. To further assess the possible effect of condition in the nest on adult morphology we evaluated nestlings’ within-brood rank as well as nestlings’ weight as predictors of their first-year adult ornament morphology (measured about a year after fledging). Within-brood rank was calculated based on nestling weight per brood; thus a nestling with the highest weight in its brood was ranked 1. When nestlings in the same brood had the same weight (differed by less than 0.2g) they received the same average rank (e.g. two nestlings in a brood that were ranked second received a rank of 2.5).

### Examining Age effects

We used two different methods to examine the effect of age on male ornament expression. First, we obtained data from successful recruitment of young individuals to the breeding population (males that were ringed as nestlings between the years 2007–2009). Those could be compared to birds known to be at least two years of age or older (adults which were ringed in previous years). However, because such an analysis may be biased when local recruits and immigrants differed in phenotype, see [56], we also examined the effect of age through changes in ornament expression across consecutive years.

### Statistical analysis

Statistical analyses were done in JMP 10 (SAS Institute), or using SPSS version 21 (IBM). We used *t-tests* or unequal variance *t-tests* to detect differences between age groups (the distribution of the examined traits did not deviate from normality). To assess father-son similarity we used mixed models incorporating nestling weight at the nest, or nestling rank at the nest (to control for certain parental non-genetic effects), together with nestling age (1 or 2 years) and father trait expression as the predicting variables. Father ID was incorporated as a random effect, thus controlling for young that had the same biological father but were raised in different years or by a different mother. Overall 22 biological fathers fathered the recruited male offspring. Finally, son ID was incorporated as a random effect to control for repeated measures of the same individual. Following lack of significant effect of the two measures of parental non-genetic effects, we removed these factors from the final models. Thus the final mixed models incorporated father trait expression and nestling age as the predictors, father ID as a random effect, son ID for repeated measure, and son trait expression as the dependent variable. Although excluded from the final model, we report the effect of within brood rank or nestling weight on male recruit morphology using Spearman rank correlation or simple linear regression, respectively.

To select between candidate models that use different predictors of sons’ trait expression (Fathers’ traits or their relative expression index) we compared their goodness of fit using Akaike’s Information Criterion correcting for small sample size (AICc) and calculated Akaike’s weights to compare their relative likelihood [57].

## Results

### Trait correlations

Within individuals, tail streamer length and ventral coloration were not significantly correlated, i.e. males with long streamers do not necessarily have dark or light ventral coloration or vice versa, as if they are genetically linked (not for fathers; *R* ^*2*^ = 0.04, *n* = 22, *P >* 0.34, or sons; *R* ^*2*^ = 0.08, *n* = 27, *P >* 0.15, or generally in the population: Figure1: *R* ^*2*^ = 0.0007, *n* = 200, *P >* 0.23 [33]. This lack of correlation between traits was also apparent when examining within-individual changes in trait expression between successive years (i.e. second year measurement—first year measurement; *R* ^*2*^ = 0.02, *n* = 46, *P >* 0.34), suggesting that an increase in the expression of one trait was not associated with either an increase or a decrease in the expression of the other trait.

### Within-individual repeatability over time

We found a significant between-year repeatability in male tail streamer length (*R* ± *SE*:*R* = 0.67 ± 0.07, *P* < 0.0001, *n* = 118, *n*_*0*_ (number of measurements per individual) = 2), male ventral coloration (*R* = 0.54 ± 0.1, *P* < 0.0001, *n* = 96, *n*_*0*_ = 2), and male relative expression index of tail streamer length and ventral coloration (expressed by the minimal distance from the diagonal line of equal expression; *R* = 0.67 ± 0.07, *P* < 0.0001, *n* = 94, *n*_*0*_ = 2). Thus, in both ornaments, the absolute level of expression as well as the relative expression index are repeatable within individuals across years and, therefore, potentially (but not necessarily) heritable.

### Father-son regression

**Fig 2:**
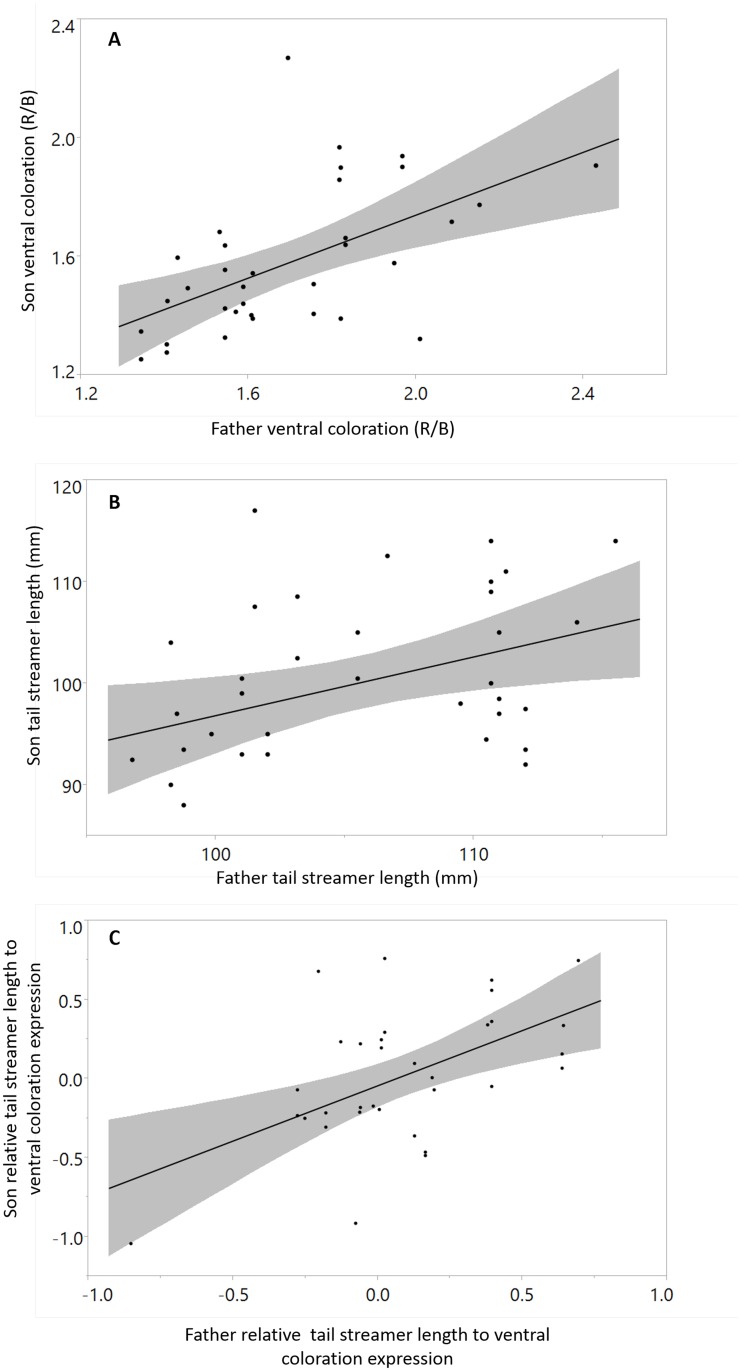
Father-son relationship of signal expression: (A) ventral coloration, (B) tail streamer length, and (C) relative expression index. Lines represent a linear fit to the scatter plot, grey area represents linear fit confidence interval, see Table 1 for details on slope and fit significance.

Father—son analysis revealed a significant relationship between ornament expression of fathers and their sons for both adult ventral coloration and streamer length (Fig. 2A and 2B; Table 1). A stronger and much more significant relationship between fathers and sons was found in the relative expression index of tail streamer length to ventral coloration (Fig. 2C; Table 1). These results remain the same also when a simpler statistical model was applied where each son is represented only by first year measurements (i.e. no repeated measurements and age effect: see Table B, Table C in S1 File).

**Table 1:** The relationship between the expression of tail streamers, ventral coloration, and their relative expression index (REI) in fathers and sons (Mixed Models with age and father trait as predictive variables, and father ID and son ID as random effects, see details in the Methods section).

*Son’s Trait expression*	Predictive variables	*n* *	*F*	*Slope* ± SE	*P*	*AICc*
Ventral coloration (VC)	Father’s VC	25	6.96	0.44 ± 0.16	**0.015**	
	Son’s age	25	0.14		0.72	
Tail streamer length (TSL)	Father’s TSL	27	5.03	0.6 ± 0.26	**0.038**	
	Son’s age	27	50.3		**< 0.001**	
TSL to VC index (REI)	Father’s REI	25	15.44	0.78 ± 0.19	**< 0.001**	23.929
	Son’s age	25	14.634		**0.004**	
	Father’s TSL	25	4.619		**0,043**	30.412
	Father’s VC	25	8.607		**0.008**	
	Son’s age	25	14.187		**0.005**	

*AICc* scores are provided for the two models that are comparable.

* 26 sons were sampled in their first year and 15 at the age of two years of which 12 were sampled in both years. Significant *P* values are marked in bold.

The strong relationship between father-son relative expression index may be explained as a by-product of father-son similarity in the expression of each trait separately. Alternatively, the heritability of each of the two traits (streamer length and ventral coloration) may be explained, in whole or in part, as a by-product of a heritable strategy for relative expression of the two traits. To evaluate the relative likelihood of these competing explanations, we first compared the Akaike’s information criteria (AICc) of the two alternative models that explain sons relative expression index (bottom of Table 1): In the first model, the predictor of son’s index is the father’s index, while in the second model, the predictors are the two separate traits of the father. This comparison showed that the second model had a higher AICc value (Table 1, ∆AICc = -6.483) giving an Akaike’s weight of 0.039, and implying that it is only 0.039 as probable as the first model (where son’s index is predicted by father’s index). This conclusion is even stronger (Akaike’s weight of only 0.0053) when the same analysis is carried out with the simpler models that are based only on one year old sons data (Table C in S1 File).

To further evaluate the relative likelihood of the competing explanations described above, we compared whether each son’s traits separately (tail length or ventral coloration) is better predicted by the same trait in the father or by the father’s relative expression index (Table 2). Indeed, stronger relationships than those found between father and son streamer length, and between father and son ventral coloration, were found between the expression of these signals by sons and the relative expression index of these signals by their fathers (Table 2). This intriguing result was especially pronounced in the case of tail streamer length (see Table 2, ∆AICc = -7.72, Akaike’s weight = 0.021), implying that a father’s relative expression index is a much better predictor of the streamer length of his son than the father’s streamer length (see Discussion).

**Table 2:** Comparing statistical models for sons’ signal expression (tail streamer length and ventral coloration) in relation to fathers’ signal expression and in relation to fathers’ relative expression index (REI), (Mixed Model with age and father trait as predictive variables, and father ID and son ID as random effects, see details in the Methods section)

*Son’s Trait expression*	Predictive variables	*N*	*F*	*P*	*AICc*
Ventral coloration (VC)	Father’s VC	25	6.96	**0.015**	-6.872
	Son’s age	25	0.14	0.72	
	Father’s REI	25	8.51	**0.008**	-7.589
	Son’s age	25	0.26	0.62	
Tail streamer length (TSL)	Father’s TSL	25*	4.21	0.056	220.978
	Son’s age	25	50.17	**< 0.001**	
	Father’s REI	25	9.44	**0.005**	213.258
	Son’s age	25	54.75	**<0.001**	

* Two males that lack data on ventral coloration (and therefore could not have relative expression index) were removed from this analysis to allow a comparison of statistical models that are based on the same individuals and sample sizes.

We found no significant evidence for a possible effect of parental care on father-son correlations, as neither nestling mass nor rank within the brood was significantly correlated with nestlings’ own adult tail streamer length or ventral coloration (*r* < 0.2, *n* = 24, *P >* 0.3, for mass; *r*_s_, < 0.1, *n = 25*, *P* > 0.6, for within brood rank). While these measures of parental care are rather crude and should be interpreted with caution, the findings are in line with previous studies conducted in Europe, which also did not find an effect of parental care on offspring’s adult tail length [28,29] but see [58] for an effect of nest sex composition on adult tail expression].

### Age effect on ornament expression

Evaluating the effect of age on ornament expression by comparing young recruits to males known to be at least two years of age or older showed that first-year males had significantly shorter tail streamers (mean ± SD = 97.3 ± 7.8 mm, *n* = 28) than older males (mean ± SD = 105.2 ± 7 mm, *n* = 46; two-sample unequal variance t-test, *t* = 4.5, *P* < 0.0001), and were significantly brighter than older males (first year males: mean ± SD = 1.57 ± 0.24 R/B ratio, *n* = 28; two year or older males: 1.75 ± 0.3 R/B ratio, *n* = 40; two-sample unequal variance t-test, *t* = 2.63, *P* < 0.01). However, we did not detect a significant effect of age on the relative ornament expression index (first year males: mean ± SD = -0.08 ± 0.07, *n* = 28; two year or older males: mean ± SD = 0.02 ± 0.06, *n* = 40; two-sample unequal variance t-test, *t* = 1.07, *P* > 0.28).

In line with the significant age effect found in the mixed models of father-son similarity (Table 1), for individual males sampled repeatedly in different years, tail streamer length increased significantly between successive years (paired t-test; mean difference = 4.31 mm, *t* = 7, *n* = 58, *P* < 0.0001), but no such significant increase was found for ventral coloration (paired t-test; mean difference = -0.0179 R/B ratio, *t* = -0.42, *n* = 48, *P* > 0.67). Within-individual change in the relative ornament expression index demonstrated a significant but slight increase towards higher expression of tail streamer length (paired t-test; mean difference = 0.14, *t* = 3, *n* = 47, *P* < 0.003). We attribute this change to the within individual increase in tail streamer length. This is because tail streamers demonstrate a highly significant change of 0.5 standard deviations while the relative expression index changes by only 0.27 standard deviations. This trend is also apparent in the results found in the mixed model analysis (see the stronger age effect of tail streamer in Table 1).

## Discussion

In this study we explored genetic and environmental determinants of sexual signal expression in the East-Mediterranean barn swallow, a population of barn swallows in which females prefer males that simultaneously express both exaggerated forms of streamer length and ventral color [32]. We examined father-son resemblance in tail streamer length and ventral coloration as well as father-son resemblance of the relative expression of these two traits as a possible indication of genetically variable advertising strategies for resource allocation between signals. As expected for sexually selected traits (either handicap or Fisherian signals [59]), we found that both signals demonstrate a significant father-son resemblance and that the expression of each signal is also affected by age. Interestingly, however, the strongest similarity between fathers and sons was found in the relative expression index of the two signals.

It is possible that when two signals are heritable, their relative expression index will also be heritable as a statistical byproduct of these primary relationships. However, in this case the heritability of this index should not be greater than that of the two signals, and a model testing the relationship between the two traits in the father and the relative expression index in the son should not give a relative likelihood weight of only 0.039 (see Results). The lack of inter-correlation between signal expression (i.e. different individuals in the population exhibited different combinations of tail length and ventral coloration; see Results), also suggests that father-son similarity in the relative expression index is unlikely to be a statistical by-product. Moreover, the result that is most difficult to explain as a statistical by-product of signal heritability is the relationship between father index and son signals, which was stronger than that between signal expression in fathers and sons (Table 2). This result implies that combining a signal *A* with another (uncorrelated) signal *B* results in an ‘*AB*’ index that is better than the fathers’ *A* itself in predicting *A*’s expression by sons. It should be clear, however, that this better predictive power of the ‘*AB*’ index cannot be attributed to the heritability of *A*. To explain this result, we must assume that the data on the *B* signal added some information about the future expression of the *A* signal by sons, which means that the expression of *A* is not independent of the expression of *B*. Note that this dependency is exactly what one should expect from the idea of genetic strategies for resource allocation between signals. According to this idea, the relative expression index should predict the future expression of each signal simply because it represents the mechanism that allocates resources to each signal and therefore determines it size. This view can also explain why the relative expression index shows a closer father-son resemblance than each of the two signals.

The possible existence of resource allocation strategies between signals can be tested further by any study on the heritability of multiple sexual signals, or even by re-analyzing existing data. All that is needed is to add a parent-offspring analysis of the relative expression of the two signals, as we did here. We hope that our study will encourage further work in this direction in a range of species.

Why might genetic strategies for resource allocation between signals have evolved? In barn swallows, for example, both tail streamer length and ventral coloration are related to feather growth and molt cycle. However, each bears a different cost and probably reveals different aspects of male quality. While long tail streamers bear an aerodynamic cost [35,21,24], dark ventral coloration seems to bear a physiological cost [36,60]. The use of multiple sexual signals in mate choice is well recognized as a common phenomenon across various taxa [5,9,10], but the trade-off between signals has been relatively overlooked [15]. Adaptive resource allocation between signals depends on their relative costs and benefits, which may vary across different habitats and populations. The cost of each signal is likely to be affected by a wide range of physiological and environmental factors, as well as by the animal’s own life-history parameters. For example, the aerodynamic cost of tail streamers for a barn swallow is almost certainly affected by the type and amount of flight that it uses for foraging and migration, which may differ considerably between the resident East-Mediterranean and the migratory European populations. The benefit of each signal is obviously affected by female preference, which may also vary within and between populations [5,61,9,10]. Thus, in each population or sub-species a different strategy for resource allocation may be selected.

It is important to note that having a genetic mechanism for resource allocation between signals may not necessarily produce high heritability of this trait. Heritability is expected only if the resource allocation strategy is also genetically variable. It is possible that in some species or populations, a single solution for resource allocation is strongly selected, which may reduce heritable variation. Thus, our study suggests that in the East-Mediterranean barn swallow, resource allocation between signals is not only genetic, but also genetically variable. What maintains this genetic variation? Among the barn swallows of our region the East-Mediterranean population is apparently unique in demonstrating a female preference for high expression of both tail streamer length and dark saturated ventral coloration [33,32]. This explains why East-Mediterranean males may be selected to allocate resources to both signals, but cannot explain why such resource allocation is genetically variable. However, considering that both the European and Egyptian barn swallow males express strongly only one signal (either long tail streamers or dark ventral coloration, respectively), and that the three populations diverged fairly recently [62] and have maintained gene-flow between them [34], genetic variability in the relative expression of the two signals is to be expected.

While further research is needed, our results to date suggest that genetically-controlled resource allocation strategies may play a significant role in the evolution of multiple sexual signals. This can be studied by monitoring the relative expression of signals and by assessing their heritability.

## Supporting Information

S1 File Figure A, Graphic illustration of hypothetic two traits which are perfectly heritable in their relative signal expression but show no heritability in their absolute level of expression. A) The expression of two uncorrelated traits (signals) by fathers (**blue**) and their sons (**red**). Father-son pairs are denoted by the same symbol and have the same distance from the diagonal line of equal relative expression, so that relative expression is perfectly heritable (see figure D). Nevertheless, despite identical relative expression strategies, the absolute magnitude of signals’ expression vary across generations (possibly due to environmental conditions) resulted in no apparent heritability of each trait (figures B and C). **Table A, Hypothetic two traits which are perfectly heritable in their relative signal expression but show no heritability in their absolute level of expression. Table B, The relationship between the expression of tail streamers, ventral coloration, and their relative expression index in fathers and their** **one year old sons**. **Table C, Two competing models testing** **one year old sons** **relative expression index (of tail streamers length TSL and ventral coloration VC) in relation to 1) their fathers relative expression index and 2) their fathers TSL and VC separately**.
(PDF)
