# Supplementary material for: Expression of Multiple Sexual Signals by Fathers and Sons in the East-Mediterranean Barn Swallow: Are Advertising Strategies Heritable?
Source: PLoS One. 2015 Feb 13;10(2):e0118054. doi: 10.1371/journal.pone.0118054 (PMC4332686; doi:10.1371/journal.pone.0118054)
Supplement: S1 File — A) The expression of two uncorrelated traits (signals) by fathers (blue) and their sons (red). Father-son pairs are denoted by the same symbol and have the same distance from the diagonal line of equal relative expression, so that relative expression is perfectly heritable (see figure D). Nevertheless, despite identical relative expression strategies, the absolute magnitude of signals’ expression vary across generations (possibly due to environmental conditions) resulted in no apparent heritability of each trait (figures B and C). Table A, Hypothetic two traits which are perfectly heritable in their relative signal expression but show no heritability in their absolute level of expression. Table B, The relationship between the expression of tail streamers, ventral coloration, and their relative expression index in fathers and their one year old sons. Table C, Two competing models testing one year old sons relative expression index (of tail streamers length TSL and ventral coloration VC) in relation to 1) their fathers relative expression index and 2) their fathers TSL and VC separately. (PDF) [file pone.0118054.s001.pdf]

Supplementary Information

The following numerical example of five individuals (Table A) demonstrates through figures Aa to Ad, how two traits (signals) may be perfectly heritable in their relative signal expression but show no heritability in their absolute level of expression. Relative signal expression was calculated as in our relative expression index (see Methods), based on the minimal distance from the diagonal line of “equal” relative expression (see figure A).

Table A

Fathers' trait 1	Fathers' trait 2	Fathers' relative expression index	Sons' trait 1	Sons' trait 2	Sons' relative expression index
5	20	10.6	18	33	10.6
31	31	0	13	13	0
25	15	-7	11	1	-7
5	25	14.1	17	37	14.1
46	31	-10.6	20	5	-10.6

Figure A a-d: a) The expression of two uncorrelated traits (signals) by fathers (**blue**) and their sons (**red**). Father-son pairs are denoted by the same symbol and have the same distance from the diagonal line of equal relative expression, so that relative expression is perfectly heritable (see figure d). Nevertheless, despite identical relative expression strategies, the absolute magnitude of signals’ expression vary across generations (possibly due to environmental conditions) resulted in no apparent heritability of each trait (figures b and c).

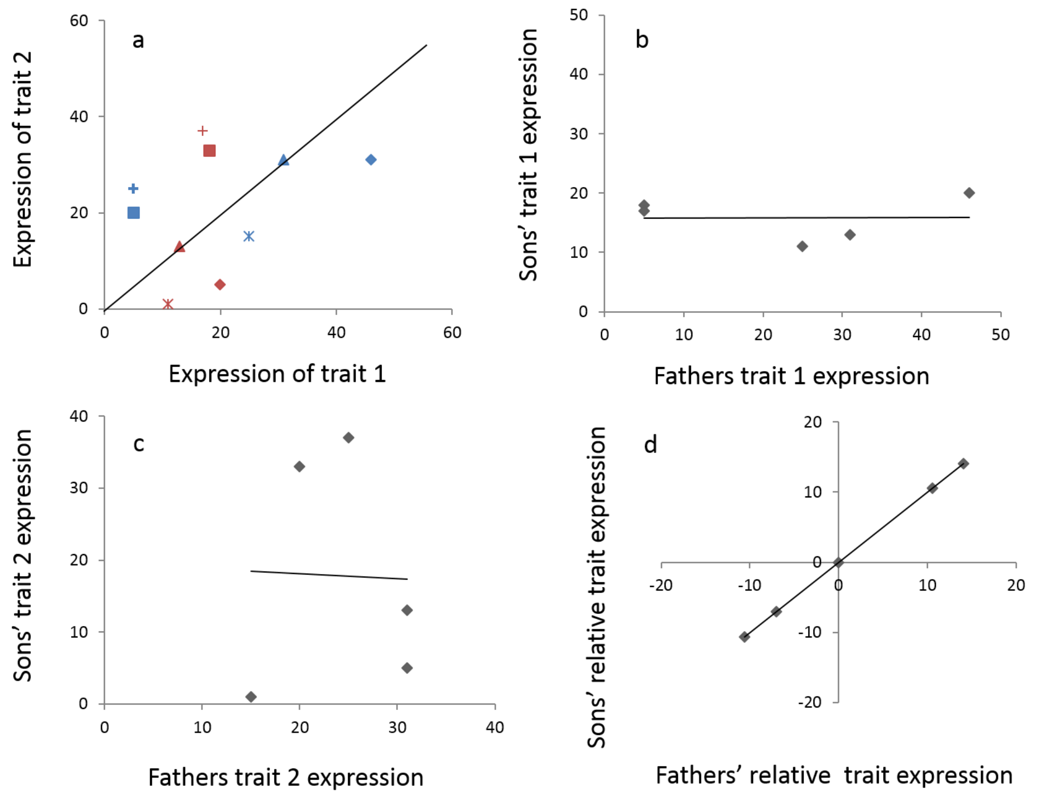

Table B: The relationship between the expression of tail streamers, ventral coloration, and their relative expression index REI in fathers and their one year old sons. This analysis takes a simpler statistical approach then in Table 1 of the paper, avoiding the need to incorporate sons age and sons repeated measure. Father ID was incorporated as random effect. These results further support our main finding: the most significant relationship between fathers and sons is in their relative expression index (see Table C below for a maximum likelihood approach).

Son's Trait expression	**Predictive variables**	***n*** *****	***F***	***Slope*** **± SE**	***P***
Ventral coloration (VC)	Father's VC	22	13.2	0.62 ± 0.17	**0.007**
Tail streamer length (TSL)	Father's TSL	24	6.3	0.67 ± 0.26	**0.022**
REI	Father's REI	22	33.1	0.89 ± 0.15	**0.0004**

Table C: The two competing models testing one year old sons relative expression index REI, (of tail streamers length TSL and ventral coloration VC) in relation to 1) their fathers REI and 2) their fathers TSL and VC separately. Lower *AICc* score indicate the more probable model. The difference between scores gives: ΔAICc = -10.47, and accordingly an Akaike's weight of 0.0053, which implies that model 2 is only 0.0053 as probable as model 1.

Son's Trait expression	**Model**	**Predictive variables**	***n***	***F***	***Slope*** **± SE**	***P***	***AICc***
Relative expression index (REI)	1	Father's REI	22	33.1	0.89 ± 0.15	**0.0004**	**17.98**
	2	Father's TSL	22	12.9	0.02 ± 0.007	**0.03**	28.45
		Father's VC	22	16.8	-0.98 ± 0.007	**0.003**	
